# Iridoids, Flavonoids, and Antioxidant Capacity of *Cornus mas*, *C. officinalis,* and *C. mas* × *C. officinalis* Fruits

**DOI:** 10.3390/biom11060776

**Published:** 2021-05-21

**Authors:** Svitlana Klymenko, Alicja Zofia Kucharska, Anna Sokół-Łętowska, Narcyz Piórecki, Dominika Przybylska, Olga Grygorieva

**Affiliations:** 1 Department of Fruit Plants Acclimatisation, M.M. Gryshko National Botanical Gardens of Ukraine National Academy of Sciences, 01014 Kyiv, Ukraine; cornusklymenko@gmail.com (S.K.); olgrygorieva@gmail.com (O.G.); 2 Department of Fruit, Vegetable and Plant Nutraceutical Technology, Wrocław University of Environmental and Life Sciences, 51-630 Wrocław, Poland; alicja.kucharska@upwr.edu.pl (A.Z.K.); anna.sokol-letowska@upwr.edu.pl (A.S.-Ł.); 3 Arboretum and Institute of Physiography in Bolestraszyce, 37-722 Przemyśl, Poland; narcyz360@gmail.com; 4 Institute of Physical Culture Sciences, Medical College, University of Rzeszów, 35-959 Rzeszów, Poland

**Keywords:** *Cornus* *mas*, *C. officinalis*, *C. mas* × *C. officinalis*, iridoids, flavonoids, antioxidant capacity

## Abstract

The fruits of *Cornus mas* and *Cornus officinalis* have been known and appreciated in folk medicine for years and have a high biological value, which is mainly connected with their polyphenols and iridoids content. However, hybrids of *C. mas* × *C. officinalis* have not been investigated. The aim of this study was to evaluate the iridoids, anthocyanins, and flavonols content, and antioxidant capacity of *Cornus mas*, *Cornus officinalis*, and *C. mas* × *C. officinalis*. Iridoids and flavonoids were quantified by the High-Performance Liquid Chromatography (HPLC) method. Antioxidant capacity (AC) was measured using 2,2-diphenyl-1-picrylhydrazyl (DPPH^•^), 2,2′-azino-bis (3-ethyl benzothiazoline-6-sulfonic acid (ABTS^•+^), and ferric reducing antioxidant power (FRAP) tests. Total phenolic content (TPC) was evaluated using the Folin–Ciocalteu reagent. Among the *C. mas* cultivars and *C. officinalis* genotypes, there was considerable variation in the content of iridoids, flavonoids, and AC. Interspecific hybrids *C. mas* × *C. officinalis* contained more iridoids than *C. mas* and more anthocyanins than *C. officinalis* and additionally had higher AC and TPC than *C. officinalis* and most *C. mas*. AC, TPC, and the presence of iridoids, anthocyanins, and flavonols in hybrids *C. mas* × *C. officinalis* are reported for the first time. The *Cornus* species deserve special attention due to their highly biologically active substances, as well as useful medicinal properties.

## 1. Introduction

*Cornus mas* L. (cornelian cherry) has been known in garden cultivation for 4000 years. This species originates from the Caucasus and from there it spread through Turkey, Romania, Bulgaria, and further on European continent [1,2]. A very rich composition of biologically active compounds found in the fruits of *Cornus mas* [3,4,5,6,7,8,9,10], which have a wide range of pharmacological action, such as antibacterial [7,11,12], hypolipidemic [7], antioxidant [6,13], anticoagulant [14], antiparasitic [15], cardioprotective [16], anticancer [17], hepatoprotective [18], and anti-inflammatory [19], and according to [20], could constrain the incidence of long-term complications of diabetes mellitus. Biologically active compounds are not only in fruits, but also in flowers, leaves, bones, and bark. The use in folk medicine of these parts of the *C. mas* plant has been known and appreciated for many years, especially in Asia [4,8,21,22,23,24,25,26,27,28,29]. Recent studies have proven that *C. mas* stones are a good source of various bioactive hydrolyzable tannins and show high antioxidant activity [30], while *C. mas* leaf extract contains a hydroxycinnamic acid derivatives, flavonols, ellagitannins, as well as iridoids, and shows high antibacterial activity against Gram-negative bacteria [31]. Moreover, latest research embraced evaluation of the unripe fruits towards the detection and isolation of biosynthetic precursors of compounds found in mature fruits [32].

Another one is no less known as a medicinal plant *Cornus officinalis* Torr. ex Dur. (Japanese cornel), which is well distributed in the Tianmu Mountain, Funiu Mountain, and Qinling-Bashan Mountain in China [33] as well as Korea and Japan [34], is virtually unknown in Europe and grows only in the collections of Botanical Gardens. It is cultivated in large areas in Japan, the local name being sandzaki. In China, *C. officinalis* is used as traditional Chinese medicine [34,35]. The fruit of *C. officinalis* is a primary medicine that was first recorded in Shen Nong’s Materia Medica about 2000 years ago [34] but was included in the Chinese Pharmacopoeia in 1963. More than 20 prescriptions containing *C. officinalis* have been used to treat various hepatic and renal diseases in China [36].

Modern pharmacological studies have shown that *C. officinalis* exhibits a broad range of pharmacological activities against diseases, such as diabetes [34,37,38,39], and has cardioprotective [40], antioxidative [41,42], anti-inflammatory, antiaging [40], neuroprotective [40,43], antibacterial [40], anti-allergic [42], and antidepressant [44] effects.

About 300 chemical compounds, such as alkaloids, iridoids, flavonoids, polysaccharides, organic acid, terpenoids, essential oils, and other compounds, have been isolated and identified from *C. officinalis* fruits [45,46,47,48,49,50,51]. The main active iridoids of *C officinalis* are morroniside and loganin. These compounds have similar hypoglycemic, nephroprotective, and neuroprotective activity. In addition, morroniside exhibits myocardial protection and antioxidant capacity [50]. Biologically active compounds are present not only in fruits but also in different parts of the plant: leaves [2,52,53], seeds [54,55,56], and twigs [2,56]. As far as *C. officinalis* is concerned, most research is devoted to pharmacological, often biochemical properties of fruit and vegetative organs, and limited data exist on breeding and culture. There are some cultivars created in Japan. For the collection of raw materials of *C. officinalis*, plantings from genotypes selected from seedlings of wild and cultivated samples are used.

There are virtually no reports about *C. mas* and *C. officinalis* crossbreeding. Information concerning their cultivation abroad is rather scant. However, Polish authors [57] described the crossbreeding hybrid from Germany, investigated its origin at the molecular level, and showed its hybridogenic origin.

In Ukraine, there are widespread cultivars of *Cornus mas* with large fruits but with lower content of some biologically active compounds. In Asia, *Cornus officinalis* is widely distributed. It has smaller fruits but high content of biologically active compounds. Probably, it is possible to select genotypes with larger fruits and much higher content of biologically active compounds by inter-hybridization of genotypes, which was the purpose of this study. According to our knowledge, this is the first detailed paper about iridoids and flavonoids content and antioxidant capacity of *C. mas × C. officinalis* hybrids. These novel hybrids could combine the beneficial features of both *C. mas* and *C. officinalis*, thus improving the composition of bioactive compounds and reducing the shortcoming of smaller *C. officinalis* fruits. In this study, *Cornus mas* and *Cornus officinalis* were used for comparison in order to provide thorough and comprehensive results.

## 2. Materials and Methods

### 2.1. Chemicals and Reagents

Loganic acid (LA), loganin (L) quercetin 3-O-glucoside (Q-glc), kaempferol 3-O-glucoside (Kf-glc), and cyanidin 3-O-glucoside (Cy-glc) were purchased from Extrasynthese (Genay, France). Further, 2,2-diphenyl-1-picrylhydrazyl (DPPH^•^) ferrous chloride, tripyridyltriazine (TPTZ), potassium persulfate, 2,2′-azino-bis(3-ethylbenzthiazoline-6-sulphonic acid) (ABTS^•+^), 6-hydroxy-2,5,7,8-tetramethylchroman-2-carboxylic acid (Trolox), and gallic acid were obtained from Sigma Chemical Co. (Steinheim, Germany). Methanol, acetonitrile, formic acid, and hydrochloric acid were obtained from POCh (Gliwice, Poland). All chemicals and solvents were of analytical grade.

### 2.2. Biological Material

**Figure 1:**
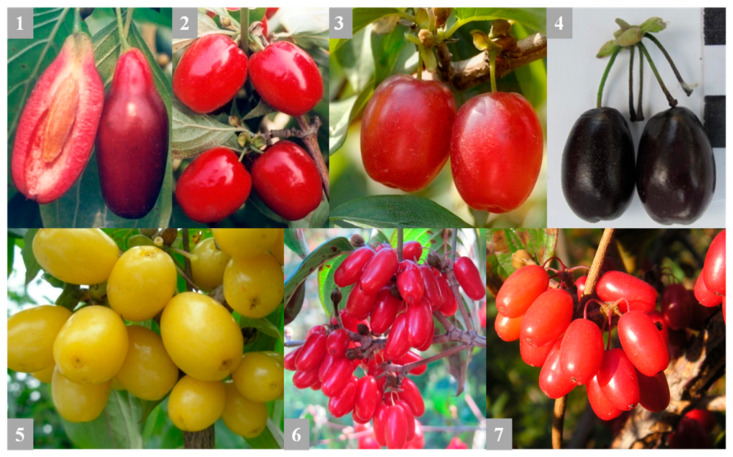
Fruits of different cultivars of *Cornus mas* L. (1—Ekzotychnyi; 2—Koralovyi Marka; 3—Koralovyi; 4—Uholok; 5—Yantarnyi), *C. officinalis* Siebold & Zucc. (6) and *C. mas* × *C. officinalis* (7) of a selection of M.M. Gryshko National Botanical Garden (Kyiv, Ukraine).

The fruits of five cultivars of *Cornus mas* (Ekzotychnyi, Koralovyi Marka, Koralovyi, Uholok (black fruit), and Yantarnyi (yellow fruit)), two genotypes *C. officinalis* (Co-01 and Co-02), and two interspecific hybrids *C. mas × C. officinalis* (CmCo-01 and CmCo-02) collected and selection in M.M. Gryshko National Botanical Garden of NAS of Ukraine (NBG) were the objects of these investigations. Plants CmCo-01 and CmCo-02 are artificial hybrids from crossing *C. officinalis* × *C. mas* [58]. The raw material was collected in the period of full ripeness (September 2015). The fruits of cultivars of *C. mas* and *C. officinalis* and *C. mas × C. officinalis* are shown in Figure 1. Approximately 500 g of fruits from 3 trees of each cultivar (approximately, 150–200 g of fruits per tree) were harvested and immediately frozen at −20 °C.

### 2.3. Preparation of Extracts for Analysis of Active Compounds and Antioxidant Capacity

Before analysis, the stones were manually removed, and the fruits without stones (300–350 g) were homogenized. The amount of about 5 g of homogenized fruits (combined from three trees for each cultivar) was extracted with 80% aqueous methanol (*v*/*v*) acidified with 1% HCl to a final volume of 50 mL at room temperature. The extraction was performed in an ultrasonic bath (Polsonic, Warsaw, Poland) for 15 min. All extracts were filtered through pickling mixture paper filters (Whatman filter No. 1, Cytiva, Marlborough, MA, USA), and then they were subjected to analyses.

### 2.4. HPLC Quantification of Iridoids and Flavonoids

The High-Performance Liquid Chromatography equipped with Photodiode Array detector (HPLC-PDA) method was described previously [59]. The quantification analysis was performed using a Dionex (Germering, Germany) system, equipped with the diode array detector model Ultimate 3000, quaternary pump LPG-3400A, autosampler EWPS-3000SI, and thermostated column compartment TCC-3000SD, and controlled by Chromeleon v. 6.8 software. The Atlantis T3 (250 mm × 4.6 mm i.d., 5 µm) column (Waters, Dublin, Ireland) and the Atlantis T3 (20 mm × 4.6 mm i.d., 5 µm) guard column (Waters, Dublin, Ireland) were used. The mobile phase was composed of solvent A (4.5% formic acid, *v*/*v*) and solvent B (acetonitrile). The elution system was as follows: 0–1 min 5% B, 1–37 min 5–25% B, 37–42 min 25–100% B, 42–47 min 100% B, 47–50 min 100–5% B, and 50–55 min 5% B. Iridoids were detected at 245 nm, flavonols at 360 nm, and anthocyanins at 520 nm. Calibration curves at concentrations ranging from 0.02 to 0.3 mg/mL (r^2^ ≥ 0.9998) were made for five standards. Iridoids were expressed as loganic acid or loganin, quercetin 3-O-glucuronide as quercetin 3-O-glucoside, kaempferol 3-O-galactoside as kaempferol 3-O-glucoside, and anthocyanins as cyanidin 3-O-glucoside. All determination were done in triplicate. The results were expressed as milligram per 100 g fresh weight (FW).

### 2.5. Determination of Total Phenolic Content (TPC) and Antioxidative Capacity (AC)

#### 2.5.1. Total Phenolic Content

Total phenolic content (TPC) of fruits was determined using the Folin–Ciocalteu reagent method according to Yen et al. [60]. Plant extracts (0.1 mL) were mixed with 0.2 mL of Folin–Ciocalteu reagent and 2 mL of H_2_O, and after 3 min, 1 mL of 20% sodium carbonate was also added. Total polyphenols were determined after 1 h of incubation at room temperature in the dark. The absorbance of the resulting blue color was measured at 765 nm. The standard curve was prepared using different concentrations of gallic acid. The results were calculated as milligram of gallic acid equivalent (GAE) per 100 g.

#### 2.5.2. FRAP Assay

Ferric reducing antioxidant power (FRAP) was measured according to Re et al. [61]. An aliquot (1.0 mL) of the diluted extract was added to 3 mL of FRAP solution (acetate buffer (300 μM, pH 3.6), a solution of 10 μM TPTZ in 40 μM HCl, and 20 μM FeCl_3_ at 10:1:1 (*v*/*v*/*v*) ratio). The mixture was shaken and left at room temperature for 10 min. The absorbance was read at 593 nm after 10 min.

#### 2.5.3. DPPH Assay

The 2,2-diphenyl-1-picrylhydrazyl free radical (DPPH^•^) scavenging capacity of fruit extracts was measured by bleaching out of the purple color of 2,2-diphenyl-1-picrylhydrazyl as described by Klymenko et al. [62]. Exactly, 0.5 mL of solution of different concentrations of the extract was added to 2 mL of DPPH ethanolic solution. The mixture was shaken and left at room temperature for 10 min. The absorbance was measured at 517 nm.

#### 2.5.4. ABTS Assay

The 2,2′-azino-bis (3-ethyl benzothiazoline-6-sulfonic acid (ABTS^•+^) assay was based on the method of Du et al. [63]. Briefly, the ABTS radical cation is generated by reacting 7 mM ABTS and 2.45 mM potassium persulfate via incubation at room temperature (23 °C) in the dark for 12–16 h. The ABTS^•+^ solution was diluted with an absorbance of 0.700 ± 0.040 at 734 nm. After the addition of 3.0 mL of diluted ABTS^•+^ solution to 30 μL of plant extracts, the absorbance reading was taken exactly 6 min after the initial mixing.

All UV–VIS measurements were recorded on a Shimadzu UV–2401PC spectrophotometer (Kyoto, Japan). All the determinations were performed in triplicate. Results of antioxidant capacity were expressed in micromole Trolox equivalent (TE) per 1 g FW. Calibration curves, in the range of 0.01–5.00 µmol Trolox L^−1^, were used for the quantification of the three methods of antioxidant activity, showing good linearity (r^2^ ≥ 0.998).

### 2.6. Statistical Analysis

Significant differences (*p* ≤ 0.05) between means were evaluated by ANOVA and the Tukey–Kramer test. Correlation coefficients were calculated using Statistica version 13.0 software (StatSoft, Tulsa, OK, USA).

## 3. Results and Discussion

In the last few years in gardening in Ukraine, Poland, as well as other countries, new fruit and berry plants, until recently encountered only in nature, have been introduced, which differ by their high maintenance of valuable biologically active substances and have important economic value. Works on the introduction and selection of new species for culture in Ukraine have been conducted in the Department of Fruit Plants Acclimatisation in NBG (Kyiv, Ukraine) for over 50 years. These species of plants include *Amelanchier* spp., *Asimina triloba* (L.) *Dunal*, *Castanea sativa* Mill., *Chaenomeles japonica* (Thunb.) Lindl. Ex Spach, *Diospyros virginiana* L., *Elaeagnus* spp., *Mespilus germanica* L., *Pseudocydonia sinensis* Schneid., *Sambucus nigra* L., *Shepherdia* spp., and *Ziziphus jujuba* Mill. [2]. Much research work is being done with *C. mas* and *C. officinalis* as food and medicinal plants [21,64].

### 3.1. Iridoids, Anthocyanins, and Flavonols of the Cornus Fruit Extracts

**Figure 2:**
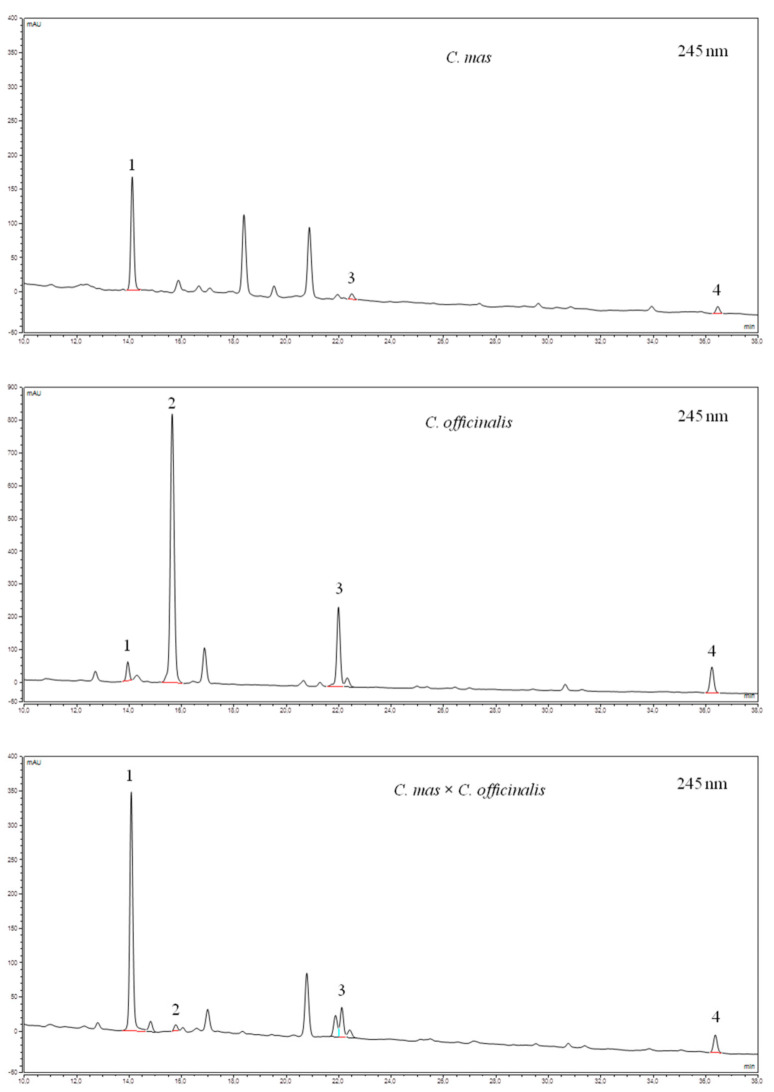
HPLC-PDA chromatograms (245 nm) of iridoids of *Cornus mas* L. (cv. Uholok), *C. officinalis* Siebold & Zucc., and *C. mas* × *C. officinalis* fruit extracts: 1—loganic acid; 2—morroniside; 3—sweroside + loganin; 4—cornuside. HPLC-PDA, High-Performance Liquid Chromatography equipped with Photodiode Array detector.

**Figure 3:**
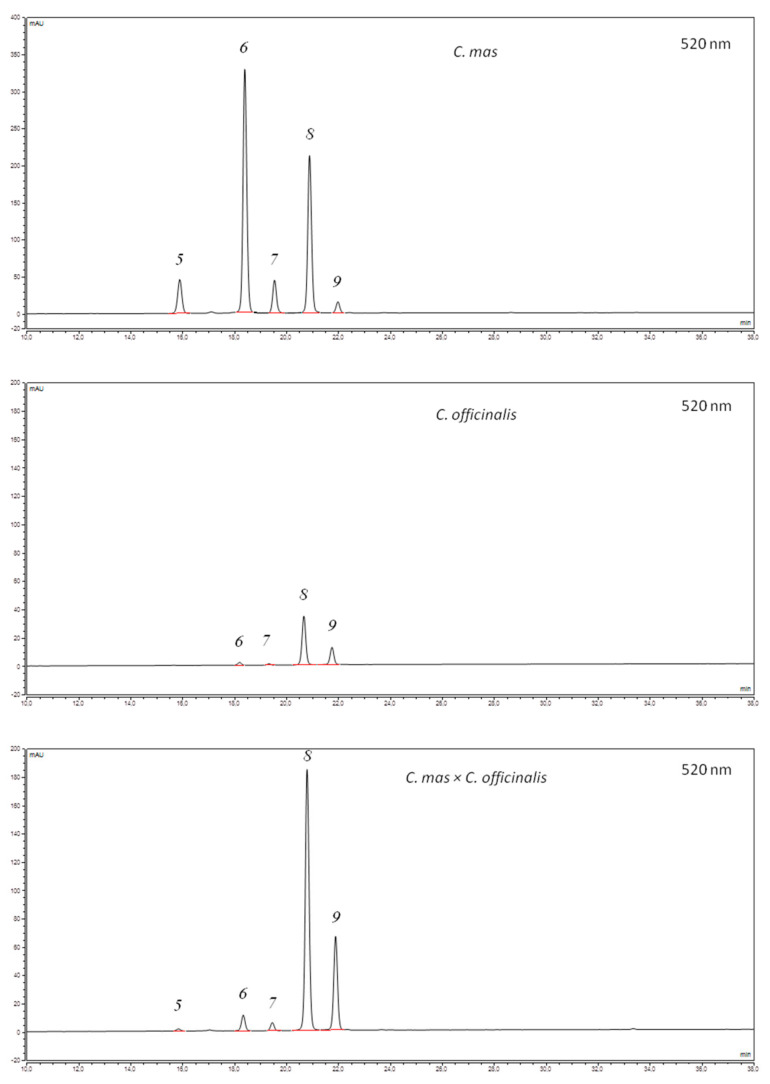
HPLC-PDA chromatograms (520 nm) of anthocyanins of *Cornus mas* L. (cv. Uholok), *C. officinalis* Siebold & Zucc., and *C. mas* × *C. officinalis* fruit extracts: 5—delphinidin 3-O-galactoside; 6—cyanidin 3-O-galactoside; 7—cyanidin 3-O-robinobioside; 8—pelargonidin 3-O-galactoside; 9—pelargonidin 3-O-robinobioside.

In the present study, iridoids, anthocyanins, and flavonols content was evaluated in 5 cultivars of *C. mas* fruits, 2 genotypes of *C. officinalis* fruits, and 2 interspecific hybrids *C. mas* × *C. officinalis* fruits (Table 1). Chromatograms of iridoids and anthocyanins of *Cornus mas* L., *C. officinalis* Siebold & Zucc., and *C. mas* × *C. officinalis* fruit extracts are shown in Figure 2 and Figure 3. The dominant group in these fruits was iridoids, followed by flavonoids including anthocyanins and flavonols.

The total iridoids content in fruits covered a wide range from 89.09 mg/100 g fresh weight (FW) (*C. mas* cv. Ekzotychnyi) to 1441.22 mg/100 g FW (*C. officinalis*, Co-01). The average iridoids contents in the analyzed *C. mas*, *C. officinalis*, and *C. mas* × *C. officinalis* fruits were 190.11, 1117.01, and 293.47 mg/100 g FW, respectively. They depended significantly both on the species and the cultivar/genotype/hybrid. *C. officinalis* fruits contained the highest amount of iridoids—almost 6 times more than *C. mas*. The iridoids in the cultivars/ecotypes of *C. mas* fresh fruits were described in a previous work [5]. The total content of the two iridoids in 26 varieties and 2 ecotypes ranged from 89.91 to 493.69 mg/100 g FW. In the case of *C. officinalis*, the content of iridoids is most often given for dried ripe fruit (*Fructus Corni*). Liu et al. [64] reported the total content of four main iridoids in *Fructus Corni* in the range of 1002–3819 mg/100 g. In our research, on average, hybrids contained 1.5 times more iridoids than *C. mas* fruit, but almost 4 times less than *C. officinalis* fruit. This is the first report to determine the iridoid content of *C. mas* × *C. officinalis* fruit.

In 5 cultivars of *C. mas* fruit, we identified loganic acid (from 77.23 mg/100 g FW for Ekzotychnyi to 239.02 mg/100 g FW for Uholok) and cornuside (from 8.81 mg/100 g FW for Koralovyi Marka to 27.07 mg/100 g FW for Uholok) and, additionally, the sum of sweroside and loganin (from 2.50 mg/100 g FW for Ekzotychnyi to 6.44 mg/100 g FW for Yantarnyi), but only in three cultivars (Ekzotychnyi, Uholok, and Yantarnyi). The dominant iridoid was loganic acid, which constituted from 87% to 96% of all iridoids. In *C. officinalis*, the iridoid profile was different than in *C. mas*. In the former, morroniside was also identified, and it constituted the largest amount (67–72% of all iridoids), while loganic acid was present in the smallest quantities (5–6% of all iridoids). Other authors [65,66] also confirmed the dominant participation of morroniside in the total amount of iridoids in *Fructus Corni*. However, Wang et al. [66] determined comparable amounts of morroniside and loganin in crude and processed *Fructus Corni* samples. The iridoid profile in hybrids was more similar to the profile of iridoids in *C. mas* fruit than to that in *C. officinalis* fruit. The dominant share of loganic acid in CmCo-01 and CmCo-02, respectively, 77% and 82% of the total amount of iridoids, indicates high similarity to *C mas*. The similarity of the hybrid to *C. officinalis* was indicated by the presence of morroniside in CmCo-01 and higher share of S + L in CmCo-01 (11%) and in CmCo-02 (12%). However, this similarity is much lower than similarity to *C. mas*.

In previous studies, it was shown that the anthocyanin content of *C. mas* fruit is within a wide range and depends mainly on the cultivar [5]. In this study, similar dependence has been found. Among cultivars of *C. mas*, low levels of anthocyanins were found in Koralovyi (4.06 mg/100 g FW) and Koralovyi Marka (33.05 mg/100 g FW), whereas Ekzotychnyi contained high amounts of colorants (74.50 mg/100 g FW, which is the typical concentration for red fruits). Black fruit of the Uholok cultivar was characterized by very high contents of anthocyanins (427.75 mg/100 g FW), and it is in this respect similar to the C27 genotype from East Azerbaijan province [27], the Czarny ecotype [5], and the Vermio cultivar—a Greek native cornelian cherry population [4]. The anthocyanin contents of Co-02 and Co-01 samples were similar at 10.52 and 17.18 mg/100 g FW, respectively. The average anthocyanin content of *C. officinalis* was over 3 times higher than the amount of anthocyanins in cv. Koralovyi, but over 2 times lower than in cv. Koralovyi Marka, over 5 times lower than in cv. Ekzotychnyi and almost 31 times lower than in cv Uholok. Vareed et al. [67] report more than 3 times lower total anthocyanin content in *C. officinalis* than in *C. mas*. For the two hybrids of *C. mas × C. officinalis*, the total content of anthocyanins ranged from 35.55 mg/100 g FW (CmCo-02) to 84.64 mg/100 g FW (CmCo-01). In our research, hybrids contained comparable amounts of anthocyanins as some cultivars of *C. mas* fruit and much higher than genotypes of *C. officinalis* fruit. This is the first report to determine the anthocyanin content of *C. mas × C. officinalis* fruit.

Five anthocyanins (delphinidin 3-O-galactoside, cyanidin 3-O-galactoside, cyanidin 3-O-robinobioside, pelargonidin 3-O-galactoside, and pelargonidin 3-O-robinobioside) were identified in most of the fruits studied. This is consistent with our previous report [5]. Among cultivars of *C. mas*, cyanidin derivatives dominated in Ekzotychnyi and Uholok fruit (redder in color), whereas pelargonidin derivatives dominated in Koralovyi Marka and Koralovyi fruits (more orange in color). In both *C. officinalis* and *C. mas × C. officinalis*, pelargonidin derivatives dominated, so the fruits were more orange than red.

The total flavonols content in *C. mas*, *C. officinalis*, and *C. mas × C. officinalis* fruit ranged from 0.47 (Koralovyi) to 10.67 mg/100 g FW (Uholok), from 8.13 (Co-02) to 12.57 mg/100 g FW (Co-01), and from 0.81 (CmCo-02) to 3.62 mg/100 g FW (CmCo-01), respectively. On average, hybrids contained similar amounts of flavonols as in *C. mas* and much lower than that in *C. officinalis*. This is the first report to determine the flavonol content of *C. mas × C. officinalis* fruit.

Quercetin 3-O-glucuronide and kaempferol 3-O-galactoside were determined in *C. mas*, *C. officinalis*, and *C. mas × C. officinalis* fruit in various proportions. The exception was the yellow fruit of the Jantarnyj cultivar, because in this sample, a derivative of kaempferol was not found. Among *C. mas*, yellow and black fruits contained significantly more quercetin 3-O-glucuronide than pink and red fruits. In the Uholok cultivar, there was almost 5 times more kaempferol 3-O-galactoside than quercetin 3-O-glucuronide, while in the Co-01 and CmCo-01 samples, there was more quercetin 3-O-glucuronide than kaempferol 3-O-galactoside, 6 times more and almost 2 times, respectively.

### 3.2. Antioxidant Capacity (AC) of Cornus Fruit Extracts

The antioxidant effect cannot be adequately tested using only one method, since plant extracts are multicomponent matrices with antioxidant capacity determined by the set of different reaction mechanisms [68,69]. Antioxidants are divided traditionally into primary and secondary ones. ABTS, DPPH, and FRAP tests provide information about direct action antioxidant (primary antioxidant). According to the mechanism of action, antioxidant tests are classified as HAT (hydrogen atom transfer) or SET (single electron transfer). In both reactions, the radical removes a hydrogen atom (HAT) or a single electron (SET) from the antioxidant and in consequence, the antioxidant becomes a radical. Distinguishing between HAT and SET mechanisms is difficult. As stated by Shalaby et al. [70], ABTS acts in HAT mode while FRAP, DPPH, and Folin–Ciocalteu tests are in SET mode, but according to Sun et al. [71], the Folin–Ciocalteu test and FRAP assay are based on the SET mechanism and ABTS and DPPH action is in mixed HAT/SET mode. In most cases, these two reactions occur simultaneously, and the reaction mechanism depends on the structure and solubility of the antioxidant, partition coefficient, and solvent polarity [71]. Therefore, for a more complete characterization of plant extracts that contain different substances, several different tests are most often performed during research [72].

In order to thoroughly evaluate the antioxidant capacity of the ethanol extracts of fruits of the studied genotypes, different antioxidant capacity assays (DPPH, ABTS, and FRAP) were employed.

**Figure 4:**
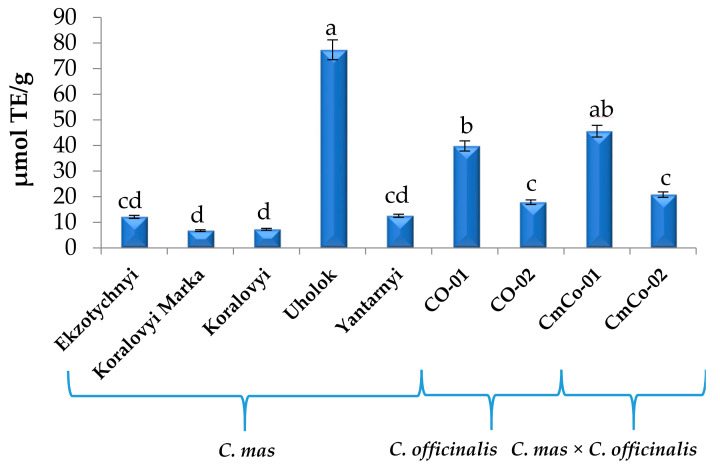
Antioxidant capacity of *Cornus mas* L., *C. officinalis* Siebold & Zucc., and *C. mas* × *C. officinalis* fruits extracts evaluated by DPPH method, μmol TE/g (means in columns followed by different letters are statistically different at *p* ≤ 0.05). DPPH, 2,2-diphenyl-1-picrylhydrazyl; TE, Trolox equivalent.

The DPPH radical scavenging capacity of each *Cornus mas* (6.75–77.35 µmol TE/g), *C. officinalis* (17.82–39.79 µmol TE/g), and *C. mas* × *C. officinalis* (20.81–45.57 µmol TE/g) extract is shown in Figure 4.

High antioxidant capacity of cv. Uholok (77.35 µmol TE/g) is evidently caused by a high content of anthocyanins in the fruits (fruits of this cultivar stand out by a very intense, almost black color). At the same time, other rose fruits of Koralovyi Marka and Koralovyi cultivars contained much less anthocyanins compared with cv. Uholok. That is probably why the AC of fruit extracts of these cultivars was the lowest, even lower than AC of extract from the yellow fruit cultivar Yantarnyi. Higher activity of yellow fruit extract than rose fruit extracts may be due to the higher content of non-red active compounds. As shown in the data in Table 1, the yellow fruits contained about 4 times more quercetin 3-O-glucuronide than the rose fruits. Antioxidant capacity of *C. officinalis* genotypes ranged from 17.82 to 39.79 µmol TE/g. It was higher than the capacity of yellow, rose, and red fruit cultivars of *Cornus mas* but lower than that of the black fruit cultivar Uholok. Antioxidant capacity of *C. mas × C. officinalis* was also high (20.81–45.57 µmol TE/g) compared with yellow, rose, and red fruit cultivars of *C. mas* because of good parent features of Lukianivsky and Olena cultivars (red fruits) from *C. mas* used in hybridization.

Many authors have studied the antioxidant capacity of *C. mas* fruit extracts. Dragovic-Uzelac et al. [73] determined that DPPH values in two different *Cornus mas* types were between 33.41 and 39.89 mmol TE/kg FW. Hassanpour et al. [27] stated that the efficiency of DPPH radicals’ scavenging depended on the total polyphenolic concentration, total flavonoids, and ascorbic acid content. In the cited study, Iranian genotypes’ fruit extracts scavenged DPPH radicals at levels varying from 38.98% to 77.6%. According to Kucharska et al. [3], antioxidant capacity in Polish breeding cultivars was the highest in the Dublany cultivar (20.72 μmol TE/g) and the lowest in the Juliusz cultivar (10.85 μmol TE/g). Fewer authors have studied the antioxidant capacity by the DPPH method of *C. officinalis* fruit extracts or products [9,74]. The IC_50_ values for DPPH radical scavenging activities of *C. officinalis* dry extracts were 99.32 μg/mL [74] and 154 μg/mL [75]. According to Lim et al. [76], the DPPH-free radical scavenging capacity of dried *C. officinalis* fruit extract (1 mg/mL) was stronger than that of vitamin E at the same concentration. West et al. [9] observed that the ability of scavenging DPPH radicals was slightly higher in the case of *C. officinalis* juice, despite the lower concentration than in *C. mas* puree.

**Figure 5:**
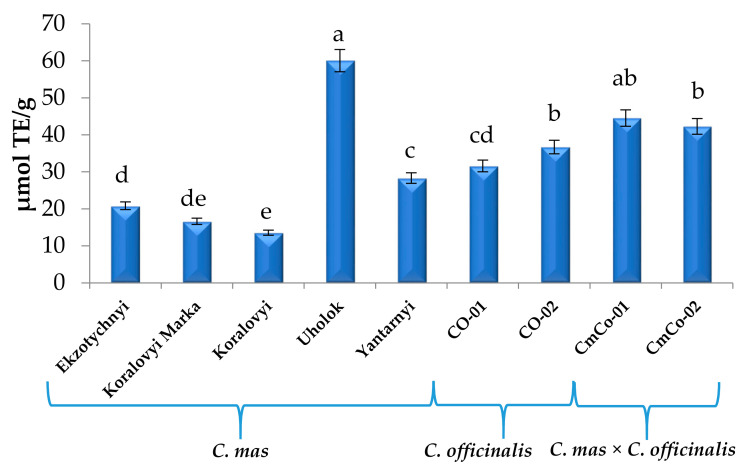
Antioxidant capacity of *Cornus mas* L., *C. officinalis* Siebold & Zucc., and *C. mas* × *C. officinalis* fruit extracts evaluated by ABTS method, μmol TE/g (means in columns followed by different letters are statistically different at *p* ≤ 0.05). ABTS, 2,2′-azino-bis (3-ethyl benzothiazoline-6-sulfonic acid.

Another stable free radical cation, ABTS, was used to evaluate the antioxidant capacity of the cornelian cherry extracts (Figure 5).

As shown in Figure 5, antioxidant capacity of extracts of *Cornus mas* cultivars measured by ABTS ranged from 13.56 (Koralovyi) to 60.04 μmol TE/g (Uholok). Antioxidant capacity by ABTS shows the same tendency that was observed in the DPPH method and also was the highest for cv. Uholok, higher in the genotypes of *C. officinalis* (31.60–36.71 µmol TE/g) than cultivars *C. mas*, and high for *C. mas × C. officinalis* (42.27–44.54 µmol TE/g), comparing all investigated genotypes (besides cv. Uholok).

Other authors obtained comparable or lower activities of *C. mas* fruits. Moldovan et al. [19] reported that the antioxidant capacity of fresh cornelian cherries was 677.88 µmol TE/100 g FW (ABTS assay). According to Rop et al. [77], the antioxidant capacity (ABTS assay) of fruits ranges from 3.65 (cv. Devin, Czech breeding) to 10.28 g of ascorbic acid equivalent per kilogram of fresh mass (AAE/kg FM) (cv. Vydubieckij, Russian breeding). According to Kucharska et al. [3], antioxidant capacity in Polish breeding cultivars of *C. mas* ranged from 18.87 (cv. Juliusz) to 38.96 μmol TE/g (cv. Szafer). *C. officinalis* antioxidant activity was measured by Hwang et al. [74]. The authors reported that the IC_50_ values for ABTS radical scavenging activities of *C. officinalis* dried extracts were 138.51 and 154 μg/mL.

**Figure 6:**
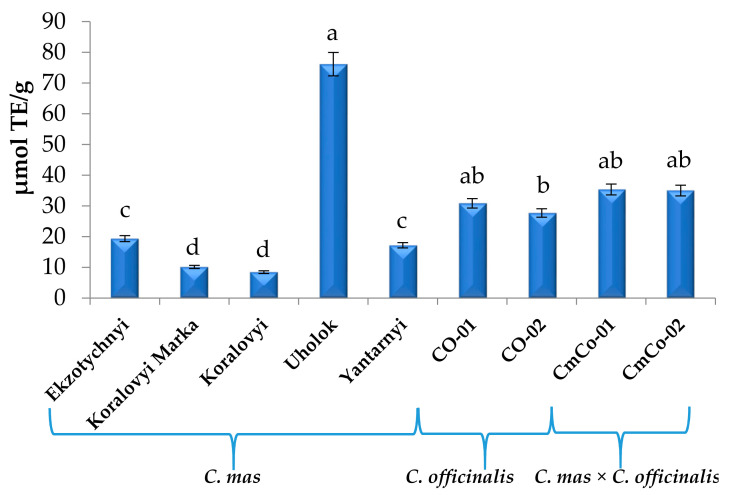
Antioxidant capacity of *Cornus mas* L., *C. officinalis* Siebold & Zucc., and *C. mas* × *C. officinalis* fruits extracts evaluated by FRAP method, μmol TE/g (means in columns followed by different letters are statistically different at *p* ≤ 0.05). FRAP, ferric reducing antioxidant power.

By analyzing the antioxidant capacity results of genotypes of *C. mas*, *C. officinalis*, and *C. mas × C. officinalis* evaluated by the FRAP method (Figure 6), one can note the same tendency as that found for its determination by the two previous methods (Figure 4 and Figure 5). Activity of hybrids *C. mas* × *C. officinalis* (35.01–35.33 µmol TE/g) was comparable to activity of *C. officinalis* (27.69–30.84 µmol TE/g) but was higher than activity of typical *C. mas* (8.45–19.34 µmol TE/g), except for Uholok (76.12 µmol TE/g).

Many authors have studied the ferric reducing antioxidant power of *C. mas*, but unfortunately, not of *C. officinalis*. Depending on the cultivars of *C. mas* tested, they obtained FRAP values within wide limits. Reducing power of Polish cultivars of *C. mas* ranges from 21.17 (cv. Juliusz) to 41.08 (cv. Szafer) μmol TE/g [3]. According to De Biaggi et al. [78], the cornelian cherry extract can reduce 20.41 μmol Fe^2+^ per gram of solution. A study conducted by Yilmaz et al. [26] presented a significant range of FRAP values, in which the minimum and the maximum values were 73 and 114 μmol of ascorbic acid equivalent per gram of dry weight (DW), respectively. Pantelidis et al. [4] confirmed that the FRAP value is approximately 84 μmol AA/g DW. In Popović’s research [13], the ferric reducing antioxidant power ranges from 2.1 to 5.8 μmol/mL Fe^2+^.

### 3.3. Total Phenolic Content (TPC) of the Cornus Fruit Extracts

**Figure 7:**
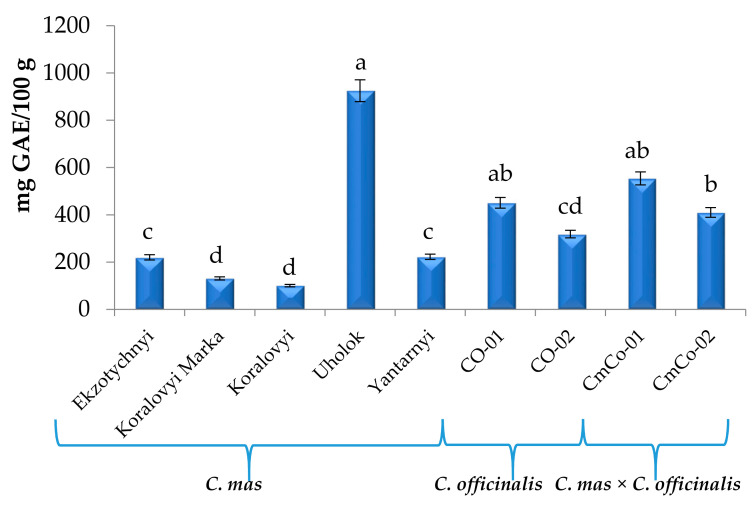
Total phenolic content (TPC) of *C. mas* L., *C. officinalis* Siebold & Zucc., and *C. mas* × *C. officinalis* fruit extracts, mg GAE/100 g (means in columns followed by different letters are statistically different at *p* ≤ 0.05). GAE, gallic acid equivalent.

The results for TPC, determined by the Folin–Ciocalteu reagent for *C. mas*, varied in a wide range from 100.71 (Koralovyi) to 924.65 mg GAE/100 g (Uholok), while for *C. officinalis* and hybrids *C. mas × C. officinalis*, the results were comparable and fell within the ranges of 318.57–451.04 and 410.20–554.01 mg/100 g, respectively (Figure 7).

The obtained results for *C. mas* were within the limits of the TPC values of *C. mas* published by several authors from Greece, the Czech Republic, Poland, Romania, Azerbaijan, and Turkey [3,6,26,28,73]. Rop et al. [77] stated that the total amount of polyphenols content was cultivar dependent. In their study, the total polyphenolic content was the lowest for cv. Devin of Czech breeding (261 mg GAE/100 g FW), while the highest concentration of polyphenol was for cv. Vydubieckii of Ukrainian breeding (811 mg GAE/100 g FW). The TPC of *Cornus mas* of Greek cultivars was 1592 mg GAE; of Azerbaijani cultivars, 1097–2695 mg GAE; and of Turkish cultivars, 2659–7483 mg GAE [26,28]. According to Cetkovska et al. [28], TPC in Ukrainian breeding cultivars was the highest in the Ekzotychnyi cultivar (614.3 mg GAE/100 g DW) and the lowest in the Lukianivsky cultivar (182.3 mg GAE/100 g DW). According to Romanian researchers, the determined TPC of the fresh ripe fruits of cornelian cherry acquired from a local market from Cluj-Napoca was 489.94 mg GAE/100 g FW [19]. Kucharska et al. [3] presented research cultivars of Polish breeding, among which the richest in polyphenols was cv. Szafer (464 mg/100 g) and the poorest was cv. Juliusz (262 mg/100 g). Polyphenol content can be linked to fruit color [79,80]. The cultivar Szafer possessed the most intense purple color of all cultivars, and it was also the richest in TPC among the cultivars tested [3]. Milenkovic-Andjelkovic et al. [29] indicated in their study that the polyphenol content slightly depended on the cultivation conditions, which differ from year to year. TPC of *C. officinalis* was measured by Hwang et al. [74] and Jeon et al. [75]. According to these authors, the TPC of *C. officinalis* dry extracts, determined by the Folin–Ciocalteu reagent, was 27.04 mg GAE/g and 34.22 mg GAE/g, respectively.

Correlation analysis was used to explore the relationships between the phenolic compounds, anthocyanins, flavonols, iridoids, and antioxidant capacities (ABTS, DPPH, and FRAP) measured for all fruit extracts from *Cornus* cultivars (Table 2).

The findings of this study indicate that TPC present high and positive correlations with FRAP scavenging capacity, DPPH, and ABTS (*r* = 0.981, *r* = 0.979, *r* = 0.942, respectively). The correlation analysis between radical scavenging and phenolic composition has been additionally proven in many studies [4,29,81].

Significant correlations were also found between the anthocyanins content and their ABTS radical scavenging (*r* = 0.716), DPPH radical scavenging (*r* = 0.843), and FRAP (*r* = 0.889) activities. The correlations between the flavonols content and antioxidant activities measured by ABTS (*r* = 0.556), DPPH (*r* = 0.707), and FRAP (*r* = 0.633) were slightly smaller than for anthocyanins and AC. Very weak correlations were observed between the iridoids content and the antioxidant activities measured by FRAP (*r* = 0.106), ABTS (*r* = 0.129), and DPPH (*r* = 0.234) assays (Table 2). This is consistent with the earlier study of Kucharska et al. [82], where it was reported that iridoids did not exhibit a high correlation between their amount and in vitro antioxidant capacity (DPPH and FRAP) of blue honeysuckle berries. However, they exhibit a number of other beneficial biological activities [83] that can complement antioxidant capacity of phenolic compounds.

These findings indicated that the TPC, anthocyanins, and flavonols, but not iridoids, are the key determinants associated with the antioxidant activities of the extracts.

## 4. Conclusions

The results of the present study showed that among the *C. mas* cultivars and *C. officinalis* genotypes, there was considerable variation in the content of iridoids and flavonoids and AC. Interspecific hybrids *C. mas × C. officinalis* contained more iridoids than *C. mas* and more anthocyanins than *C. officinalis*, and additionally, they had higher AC and TPC than *C. officinalis* and most *C. mas*. All fruit extracts of *Cornus mas*, *C. officinalis*, and *C. mas × C. officinalis* exhibited strong antioxidant activities, which correlated positively with the total phenolic content and phenolic compounds as anthocyanins and flavonols, but did not correlate with the iridoids content. In the *C. mas* cultivars, activity depended on the color of the fruit. The cultivar Uholok with almost black fruit had the highest antioxidant potential, while the pink fruit cultivars had the lowest. The AC of *C. mas × C. officinalis* new hybrids was higher than that of the AC of *C. officinalis* genotypes and *C. mas* cultivars, except for cv. Uholok. Genotypes of our selection differ in higher values of biochemical parameters in comparison with cultivars of other studies. Currently, this plant is less known and this new source can potentially be used as a functional food or value-added ingredient in the future in our dietary system. The results of our study suggest the great value of the fruits of *Cornus mas*, *C. officinalis*, and particularly of *C. mas* × *C. officinalis* for use in pharmacy and phytotherapy. Therefore, further studies should be conducted, with particular attention to validate these in vitro results in cellular or animal models.

## Figures and Tables

**Table 1:** Iridoids, anthocyanins, and flavonols content (mg/100 g FW) in *Cornus mas* L., *C. officinalis* Siebold & Zucc., and *C. mas* × *C. officinalis* fruits.

Compound ^1^	*C. mas*					*C. officinalis*		*C. mas × C. officinalis*	
	Ekzotychnyi	Koralovyi Marka	Koralovyi	Uholok	Yantarnyi	Co-01	Co-02	CmCo-01	CmCo-02
Iridoids									
LA	77.23 ± 0.83 e ^2^	156.31 ± 27.45 d	192.71 ± 15.00 c	239.02 ± 1.79 b	200.72 ± 0.34 c	70.76 ± 6.32 e,f	46.15 ± 3.14 f	269.32 ± 3.68 a	193.64 ± 16.16 c
Mo	nd ^3^	nd	nd	nd	nd	1037.74 ± 87.52 a	529.52 ± 30.02 b	9.68 ± 0.32 c	nd
S + L	2.50 ± 0.37 d	nd	nd	2.84 ± 0.25 d	6.44 ± 0.66 d	223.55 ± 18.11 a	172.16 ± 10.88 b	38.14 ± 0.72 c	28.27 ± 1.72 c
Co	9.36 ± 0.56 e	8.81 ± 1.37 e	9.04 ± 0.92 e	27.07 ± 0.86 c	18.51 ± 0.13 d	109.17 ± 9.32 a	44.98 ± 3.49 b	32.51 ± 0.27 c	15.38 ± 2.11 d,e
Total	89.09 e	165.12 d,e	201.75 d	268.93 c,d	225.67 d	1441.22 a	792.81 b	349.65 c	237.29 d
Anthocyanins									
Df-gal	0.39 ± 0.02 b	0,26 ± 0.04 b	nd	32.81 ± 0.58 a	nd	nd	tr ^4^	tr	0.69 ± 0.14 b
Cy-gal	41.04 ± 0.14 b	6.83 ± 1.18 c	0.24 ± 0.02 c	216.28 ± 8.52 a	nd	0.58 ± 0.03 c	1.20 ± 0.14 c	3.43 ± 0.13 c	1.75 ± 0.21 c
Cy-rob	7.62 ± 0.02 b	1.08 ± 0.09 c,d	nd	30.00 ± 1.83 a	nd	0.28 ± 0.04 c,d	0.35 ± 0.03 c,d	1.69 ± 0.02 c	1.08 ± 0.13 c,d
Pg-gal	23.59 ± 0.12 c	23.12 ± 3.18 c	3.82 ± 0.35 e	138.79 ± 3.16 a	nd	11.99 ± 1.20 d	6.59 ± 0.60 e	58.78 ± 1.53 b	20.79 ± 3.27 c
Pg-rob	1.86 ± 0.00 d	1.76 ± 0.17 d	nd	9.87 ± 0.51 b	nd	4.33 ± 0.43 c	2.38 ± 0.12 d	20.74 ± 0.66 a	11.24 ± 1.66 b
Total	74.50 b	33.05 c	4.06 d	427.75 a	nd	17.18 d	10.52 d	84.64 b	35.55 c
Flavonols									
Q-glcr	0.22 ± 0.01 d	0.48 ± 0.07 d	0.40 ± 0.02 d	1.85 ± 0.04 c	1.90 ± 0.07 c	10.83 ± 1.28 a	7.89 ± 0.72 b	2.39 ± 0.20 c	0.59 ± 0.05 d
Kf-gal	1.06 ± 0.04 c	0.48 ± 0.08 d	0.07 ± 0.01 e,f	8.82 ± 0.17 a	nd	1.74 ± 0.13 b	0.24 ± 0.03 e	1.23 ± 0.01 c	0.22 ± 0.04 e
Total	1.28 e,f	0.96 e,f	0.47 f	10.67 b	1.90 e	12.57 a	8.13 c	3.62 d	0.81 e,f

^1^ LA, loganic acid; Mo, morroniside; S, sweroside; L, loganin; Co, cornuside; Df-gal, delphinidin 3-O-galactoside; Cy-gal, cyanidin 3-O-galactoside; Cy-rob, cyanidin 3-O-robinobioside; Pg-gal, pelargonidin 3-O-galactoside; Pg-rob, pelargonidin 3-O-robinobioside; Q-glcr, quercetin 3-O-glucuronide; Kf-gal, kaempferol 3-O-galactoside. ^2^ Values are expressed as the mean (n = 3) ± standard deviation. Mean values with different letters: a, b, c, etc. are statistically different (*p* < 0.05). ^3^ nd, not detected. ^4^ tr, traces.

**Table 2:** Correlation coefficients between antioxidant capacity and bioactive compounds.

Components	DPPH	ABTS	FRAP
Anthocyanins	0.843 *	0.716 *	0.889 *
Iridoids	0.234	0.129	0.106
Flavonols	0.707 *	0.556	0.633
TPC	0.979 *	0.942 *	0.981 *

* Correlation is significant at the 0.05 level.
